# RegScaf: a regression approach to scaffolding

**DOI:** 10.1093/bioinformatics/btac174

**Published:** 2022-03-25

**Authors:** Mengtian Li, Lei M Li

**Affiliations:** National Center of Mathematics and Interdisciplinary Sciences, Academy of Mathematics and Systems Science, Chinese Academy of Sciences, Beijing 100190, China; University of Chinese Academy of Sciences, Beijing 100049, China; National Center of Mathematics and Interdisciplinary Sciences, Academy of Mathematics and Systems Science, Chinese Academy of Sciences, Beijing 100190, China; University of Chinese Academy of Sciences, Beijing 100049, China

## Abstract

**Motivation:**

Crucial to the correctness of a genome assembly is the accuracy of the underlying scaffolds that specify the orders and orientations of contigs together with the gap distances between contigs. The current methods construct scaffolds based on the alignments of ‘linking’ reads against contigs. We found that some ‘optimal’ alignments are mistaken due to factors such as the contig boundary effect, particularly in the presence of repeats. Occasionally, the incorrect alignments can even overwhelm the correct ones. The detection of the incorrect linking information is challenging in any existing methods.

**Results:**

In this study, we present a novel scaffolding method RegScaf. It first examines the distribution of distances between contigs from read alignment by the kernel density. When multiple modes are shown in a density, orientation-supported links are grouped into clusters, each of which defines a linking distance corresponding to a mode. The linear model parameterizes contigs by their positions on the genome; then each linking distance between a pair of contigs is taken as an observation on the difference of their positions. The parameters are estimated by minimizing a global loss function, which is a version of trimmed sum of squares. The least trimmed squares estimate has such a high breakdown value that it can automatically remove the mistaken linking distances. The results on both synthetic and real datasets demonstrate that RegScaf outperforms some popular scaffolders, especially in the accuracy of gap estimates by substantially reducing extremely abnormal errors. Its strength in resolving repeat regions is exemplified by a real case. Its adaptability to large genomes and TGS long reads is validated as well.

**Availability and implementation:**

RegScaf is publicly available at https://github.com/lemontealala/RegScaf.git.

**Supplementary information:**

Supplementary data are available at *Bioinformatics* online.

## 1 Introduction

Along with the development of high-throughput sequencing technology, various assembling methods have been developed to pursue complete, continuous and accurate genome assemblies. Whether a method is based on the de Bruijn graph or the overlap graph, *de novo* assembly usually requires three steps: (i) generating short continuous sequences called contigs; (ii) incorporating contigs into longer scaffolds; (iii) filling the gaps. The scaffolding step aims to group contigs and to infer their orientations as well as orders within each scaffold. In addition, the scaffolding procedure should give an estimate of the gap distance between adjacent contigs, together with a fair assessment of reliability, which, in our view, should be taken part in the genome quality.

Second-Generation Sequencing (SGS) paired reads are typically used to order and orientate contigs. For instance, Illumina supports two types of paired-read libraries: short-insert paired-end (PE) and long-insert mate-pairs (MP). If two paired reads are mapped to two different contigs, this read pair establishes a *link* between its two mapped contigs, which includes their linking distance as well as their relative orientations. The linking distance is a linear combination of the insert-size and the mapping positions of the paired reads on contigs; the relative orientation takes value +1 or -1 depending on whether the directions of the two contigs are consistent or not. Similarly, a long read from Third-Generation Sequencing (TGS) platform whose two different segments are mapped to two contigs also establishes a link between them. Many scaffolding methods are developed based on graphs which represent the above links between contigs. One popular method is to extract paths containing as fewer conflicts as possible from the graph, and each path corresponds to a scaffold.

The path-finding method formalizes the scaffolding problem as an optimization problem that maximizes the sum of weights of happy mate-edges (Huson *et al.*, 2002), which are defined to be consistent with the relative orientations and be compatible with the theoretical distance between linked contigs. Because of its non-polynomial time complexity, certain simplifications are necessary. Some scaffolders, such as SSPACE (Boetzer *et al.*, 2011) and the built-in scaffolding procedure in SOAP2 (Luo *et al.*, 2012), take heuristic strategies by adding the most reliable contig one by one. Some other scaffolders approximate the optimal solution by mathematical techniques such as the graph method (Huson *et al.*, 2002) and the dynamic programming method in Opera-LG (Gao *et al.*, 2011, 2016).

The major difficulty in scaffolding is the presence of incompatible linking distances and conflicting orientations within one contig subset. Although non-unique mapping reads are filtered out during preprocessing, the missing duplicate regions on preliminary contigs, either completely or partially, could lead to mistaken alignment of paired reads. Supplementary Figure S3 illustrates such a typical scenario, in which one read is mapped to the correct genomic position whereas the other is mapped to an incorrect position. The latter is usually a homologous duplicate of the real source. Links from such alignment generate misleading linking distances and wrong relative orientations between contigs. Sometimes correct links are overwhelmed by the incorrect ones. The detection of such incorrect links is challenging in any existing methods.

In this report, we present a novel scaffolding approach, referred to as RegScaf, in which pairwise contig distances, allowing multiple values, are integrated by an iterative robust regression. RegScaf first examines the distribution of linking distances between pairwise contigs by its kernel density. When multiple modes are found in a density, values corresponding to these modes, instead of a single average, are input into a linear regression model. The linear model parameterizes contigs by their positions on the genome; then each linking distance between a pair of contigs is taken as an observation on the difference of their position parameters. Consequently, scaffolding becomes a parameter estimating problem, taking the portion of incorrect linking distances as outlier observations. To ensure the estimates of the contig positions would not break down by the outliers, we adopt a robust method that minimizes a sum of trimmed squares.

The evaluation of RegScaf is carried out using several datasets. First, we test RegScaf on both synthetic datasets and GAGE (Salzberg *et al.*, 2012) benchmark datasets, and the results show that RegScaf outperforms many popular scaffolders, especially in the accuracy of gap estimates by substantially reducing extremely abnormal errors. Second, we exemplify its strength in resolving repeat regions by a real instance. Third, we demonstrate its robustness to contaminated libraries by a simulation. Fourth, we show RegScaf outperforms SSPACE in the sequencing project of the *Ochotona* *curzoniae* genome, whose size is about 2.5 Gb. Last, we prove its adaptability to TGS long reads by producing a hybrid assembly based on two Pacbio datasets.

## 2 Materials and methods

### 2.1 Overview

**Fig. 1.**
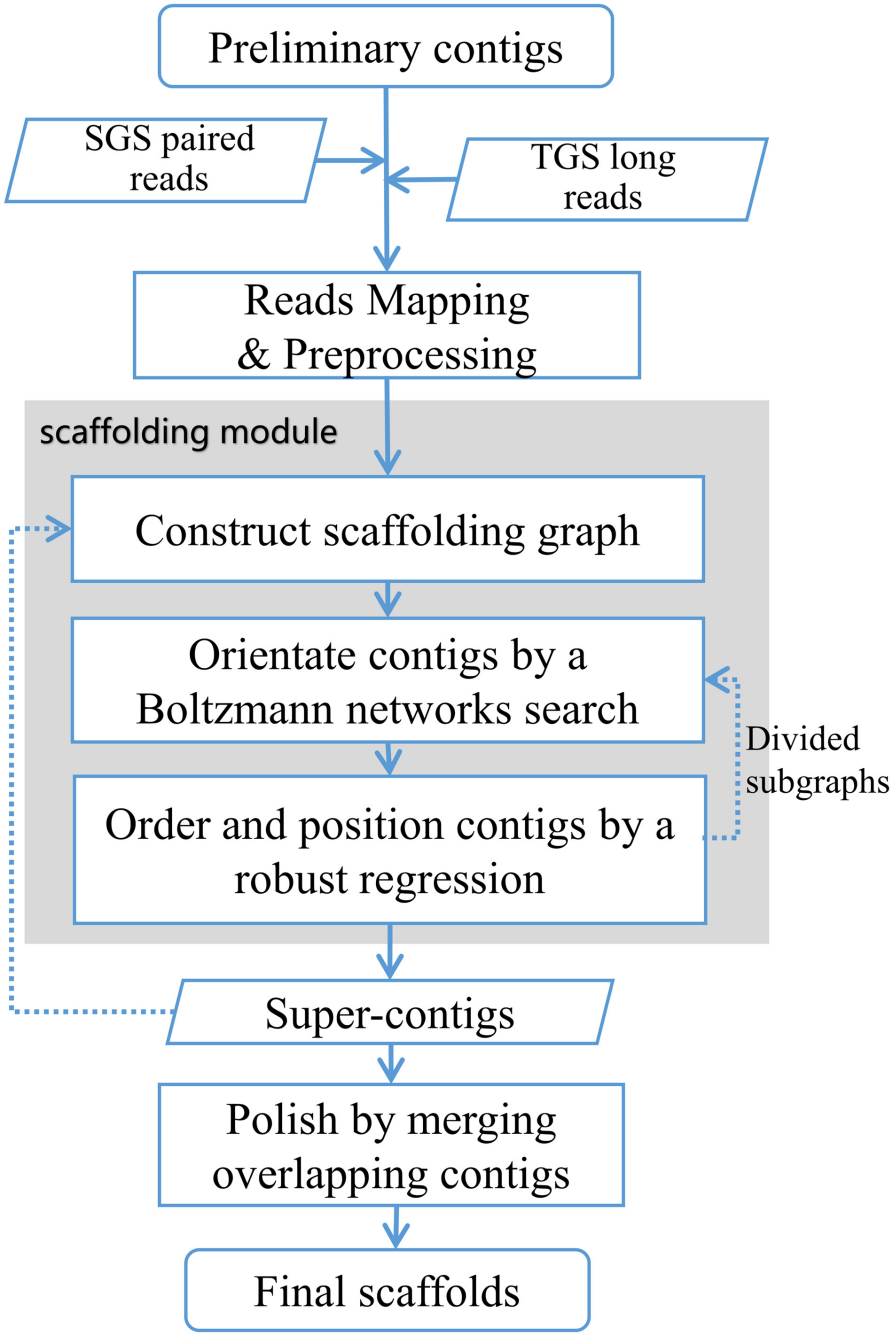
The workflow of RegScaf

The workflow of RegScaf is given in Figure 1. The input of RegScaf contains a preliminary contig assembly and sequencing reads. An initial step is the mapping of sequencing reads, either paired reads or TGS long reads, against preliminary contigs, followed by a preprocessing step. The major scaffolding module consists of three steps shown in the gray box. Step 1, construct scaffolding graph based on links and split the graph into maximal connected subgraphs, each of which provides a candidate subset of the preliminary contigs for scaffolding. Step 2, orientate contigs within each subgraph by a Boltzmann networks search. Step 3, order and position contigs within each subgraph by a robust regression method. Step 3 may break a connected component by detecting and removing unreliable links; then the divided smaller subgraphs will go through the step 2 again. The regression result will then be untangled into *super-contigs*, each of which is a linear placement of orientated contigs separated by gaps whose lengths are robustly estimated. With super-contigs in place of initial contigs, the scaffolding pipeline is iterated to generate longer and more reliable scaffolds. After *k* (a user-specified parameter) iterations, the super-contigs are polished by merging overlapping contigs before final scaffolds are output. We describe the details of each step in Sections 2.2–2.6.

### 2.2 Reads mapping and preprocessing

RegScaf integrates SEME (Chen *et al.*, 2013), a fast and accurate mapping tool, to map SGS paired reads to contigs. For TGS long reads, RegScaf integrates BLASR (Chaisson and Tesler, 2012)—the most popular mapping tool for TGS data. When preprocessing the mapping results, RegScaf first removes ambiguous reads that are mapped to multiple areas or high-coverage areas (see how to mark high-coverage area in Supplementary Note S1). RegScaf then re-estimates the insert-size *μ_b_* and its variance $\sigma_{b}^{2}$ for each library *b* based on the read pairs that are mapped to the same long contig (length >10kbp), and removes those read pairs which map too far from the contig edge, namely, beyond $\mu_{b}+3\sigma_{b}$. Some preprocessing scripts are inherited from BAUM (Wang *et al.*, 2018).

### 2.3 Constructing the scaffolding graph

We take a graph structure to group contigs into connected components, each of which serves as a candidate scaffold set. Similar to the contig connectivity graph in SOPRA (Dayarian *et al.*, 2010) or Opera-LG, the scaffolding graph is constructed by setting contigs as vertexes. To reduce noises in links, we draw an edge between two contigs only if the number of links between them exceeds a preset threshold, which is a user-specified parameter in RegScaf. Considering that noises are mainly from chimeric reads and repeat regions in a genome, we suggest users set the threshold between 0.25× Coverage and 0.5× Coverage. We also provide a default option which takes the 10% quantile of the non-zero link count distribution in RegScaf.

Then we apply a depth-first-search (DFS) algorithm to obtain all connected subgraphs of the scaffolding graph. Each subgraph corresponds to a potential subset of contigs that form a scaffold. We implement this algorithm by a recursion whose pseudo-code is shown in Supplementary Note S10. The following scaffolding procedure will be applied to all subgraphs in parallel.

**Fig. 2.**
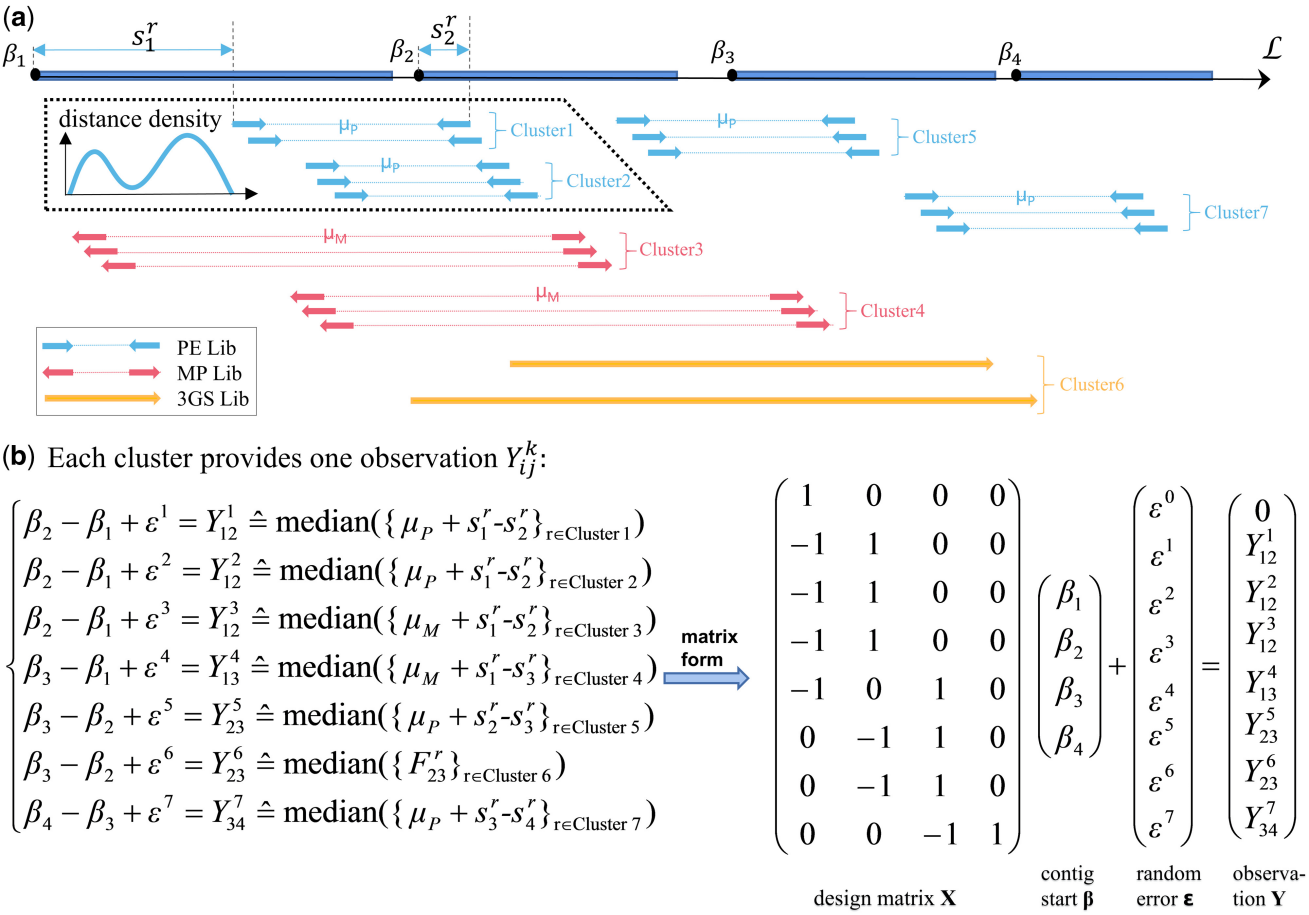
An illustration of the regression model in RegScaf. (**a**) Clustering of mapping reads according to their linking distance. At the top lies the axis L corresponding to the scaffold containing four orientated contigs, with each long box representing a contig and each black point indicating its start coordinate *β_i_* in the axis. Input reads can be any combination of the three types: PE lib (paired-end library, inward paired arrows), MP lib (mate-pair library, outward paired arrows) and TGS lib (long arrows). The sequencing reads that are mapped to two different contigs are taken as links, each of which suggests a distance calculated from the read alignment. But sometimes links suggest quite different distances for the same contig pair; then links are partitioned into several clusters according to the distance density distribution. As the example in the dotted box shows, PE links between contigs 1 and 2 are partitioned into two clusters for the distance density shows two peaks. (**b**) Each cluster represents an independent observation in the regression model. For each cluster, we take the median of all linking distances as the value of $Y_{ij}^{k}$, which represents the observed difference between the two contig start coordinates, and build the linear regression model $\beta_{j}-\beta_{i}+\epsilon^{k}=Y_{ij}^{k}$ where *ϵ^k^* indicates the error term. Seven clusters provide seven distinct observations, and we integrate them into the matrix form as shown. Notice that we add the first row: $\beta_{1}=0$, to ensure the model recognizable. For some contig pairs, such as contigs 1 and 2, multiple observations indicating different mapping modes are simultaneously retained in this model

Before we proceed, we set up some notations. Denote one of the connected contig subgraphs as **G**, with its contig vertex set $V_{G}=\{1,2,\ldots ,m\}$. We first set notations for links between contig vertexes of **G**, where a link is a SGS paired read or a TGS long read which maps to two distinct contigs, see different kinds of links in Figure 2a. Assume a read *r* maps to contigs *i* and *j*, respectively, where i=i(r) and j=j(r) depend on *r* but we omit them for the sake of simplicity. If the read *r* indicates contigs *i* and *j* have the same orientation, we set their relative orientation $d_{ij}^{r}=1$; otherwise $d_{ij}^{r}=-1$.

To represent the DNA sequence which the contigs are from, consider a directed straight axis L orientating in the direction from 5′ to 3′ end of contig 1. Under this setting, the goal of scaffolding is to get an orientation assignment $D=(1,D_{2},\ldots ,D_{m})$ and an estimate of the vector of the contig start coordinates $\beta =(\beta_{1},\beta_{2},\ldots ,\beta_{m})$, where *D_i_* takes the value +1 if the orientation of the contig *i* is forward in L and takes -1 otherwise, and *β_i_* denotes the start coordinate of contig *i* in L. To achieve this goal, we decompose the scaffolding problem into two parts: (i) the global orientating problem; (ii) the position estimation problem. The former will be solved by an optimization model in Section 2.4, while the latter will be formalized as a parameter estimation problem in a regression model in Section 2.5.

### 2.4 Orientating contigs by a Boltzmann networks search

In the orientating problem, each paired read *r* imposes a constraint on the relative orientation between two contigs: $D_{i}D_{j}=d_{ij}^{r}$. However, contradictions may appear when we pool all pairwise constraints together. We propose to represent such binary state vectors ($D_{i}=\pm 1$) along with their pairwise orientation constraints by a Boltzmann networks (Ackley *et al.*, 1985). Consequently, orientating contigs becomes a search problem in the Boltzmann networks. Specifically, we take the optimal solution that minimizes the following Hamilton energy function:

(1) $$H(D)=-\sum_{i\neq j;i,j\in V_{G}}J_{ij}D_{i}D_{j},$$

where $J_{ij}=(a_{ij}-b_{ij})/2,\;a_{ij}=\#\{r:d_{ij}^{r}=1\},\;b_{ij}=\#\{r:d_{ij}^{r}=-1\},\;\#$ denotes the number of elements in the set. Notably, despite the similarity in form, this energy function is different from that in SOPRA where $J_{ij}≜[\mathrm{sign}(a_{ij}-b_{ij})](a_{ij}+b_{ij})$. In fact, we have proved that the solution to minimizing (1) is equivalent to the maximum likelihood solution when assuming each link has an equal error probability in Supplementary Note S2.

As this combinatorial optimization problem has been proved to be NP-complete (Garey and Johnson, 1979), RegScaf adopts a heuristic algorithm, which first initializes the orientation assignment by a weight-decreasing depth-first search and then iteratively optimizes **H** node by node. Detailed algorithm can be found in Supplementary Note S3.

Once the contigs’ orientations have been determined on the current subgraph: $\hat{D}=(1,{\hat{D}}_{2},\ldots ,{\hat{D}}_{m})$, we adjust all contigs into the same orientation by reversing contigs assigned with negative ${\hat{D}}_{i}$. Next, we compute relative linking distance between contigs, in preparation for the following step that estimates contig exact positions. For each contig pair (*i*, *j*), we only consider orientation-supported links: $R_{ij}=\{r:d_{ij}^{r}={\hat{D}}_{i}{\hat{D}}_{j}\}$. Then we compute the linking distance $F_{ij}^{r}$, which indicates the difference between the contig start coordinate observed by the paired read *r*. Denote $s_{i}^{r}$ and $s_{j}^{r}$ be the mapping coordinates on the contig *i* and *j*, respectively; the mapping coordinate records the distance between the contig start and the outer end of the mapped read (see Fig. 2a). Therefore, the observed linking distance between the start coordinates of contig *i* and *j* from read *r* can be expressed as: $F_{ij}^{r}=\mu_{b}+s_{i}^{r}-s_{j}^{r}$ if contig *i* is upstream of contig *j*; otherwise $F_{ij}^{r}=-(\mu_{b}-s_{i}^{r}+s_{j}^{r})$ (Supplementary Note S4 and Supplementary Fig. S1). Computations for TGS long reads are a little different, where the linking distance can be expressed as: $F_{ij}^{r}=qs_{j}^{r}-qs_{i}^{r}+cs_{i}^{r}-cs_{j}^{r}$, where $qs_{i}^{r}$ denotes the alignment start on long read *r* and $cs_{i}^{r}$ denotes the alignment start on contig *i* (Supplementary Note S5 and Supplementary Fig. S2).

### 2.5 Positioning contigs by a robust regression

Since we have parameterized contigs by their starts on the genome: $\beta =(\beta_{1},\beta_{2},\ldots ,\beta_{m})$, each linking distance $F_{ij}^{r}$ is represented as an observation on the difference of the two parameters: $F_{ij}^{r}=-\beta_{i(r)}+\beta_{j(r)}$. Indeed, we can integrate thousands of observations on the connected subgraph into a global model, which fits the relationship between the observed linking distance (the dependent variable *Y*) and the corresponding contig indices (the explanatory variable $X=(X_{1},\ldots ,X_{m}),X_{i}\in \{0,1,-1\}$) by a linear regression:

$$Y=\beta_{1}X_{1}+\beta_{2}X_{2}+\cdots +\beta_{m}X_{m}+\epsilon .$$

The error item *ϵ* represents the random error which is mainly from the variation of library insert-sizes.

A straightforward estimate of β can be obtained using all pairwise links, each corresponding to an observation but this is computationally intensive when one subgraph contains millions of links. Alternatively, all links between each contig pair can be compressed into one single observation, by either the median or the mean of the distances. Indeed, several current scaffolders (Dayarian *et al.*, 2010; Gao *et al.*, 2016) adopt this strategy. Nevertheless, such brute force compression strategy could cause a loss of information that leads to misassemblies eventually on some occasions. One such example will be explained in Section 3.2. To trade off computational efficiency and accuracy, we adopt a multi-mode strategy. That is, we first examine the distribution of the linking distances for each contig pair: if multiple modes are shown, we group links into several clusters, each corresponding to a mode. Each cluster of links is represented by one observation weighted by the cluster size and variance. Thus the strategy, compared with existing methods, could retain multiple observations for a contig pair, and the correct one will be selected later by a robust regression procedure. Moreover, considering that insert-size distribution varies across libraries, we carried out the clustering for each library separately.

A such instance is shown in Figure 2, which includes four steps for compressing links between the contig pair (*i*, *j*) from the library *b*. First, we calculate the kernel density of their linking distance $\left\{F_{ij}^{r}:r\in R_{ij},r\;\text{in}\;\text{Library}\;b\right\}$ where the bandwidth is set to be $\sigma_{b}/2$. Second, we mark those peaks which are well separated in the kernel density and denote the number of peaks as *K_ij_*. Third, we group links into clusters by assigning each link to its nearest peak. Fourth, for each cluster $R_{i,j}^{k}$, we take the median as the compressed observation on the difference of two contig position parameters: $Y_{ij}^{k}≜\mathrm{median}(\{F_{ij}^{r}:r\in R_{ij}^{k}\})$ and fit the regression model:

(2) $$Y_{ij}^{k}=-\beta_{i}+\beta_{j}+\epsilon^{k}.$$

According to the central limit theorem of median (Shao, 1999, Theorem 5.10), we have $\epsilon^{k}∼N(0,\frac{\pi \sigma_{b}^{2}}{2n_{ij}^{k}})$, where $n_{ij}^{k}$ is the number of links in the cluster $R_{ij}^{k}$. In other word, the *k*-th observation is assigned a weight $w_{ij}^{k}=\frac{n_{i,j}^{k}}{\sigma_{b}^{2}}$. In practice, we replace $\sigma_{b}^{2}$ by the sample variance of the current cluster when the cluster size is larger than 6.

Finally, we pool together all compressed observations from different libraries and different contig pairs in the current subgraph into the matrix form (see the example in Fig. 2b), where we set $\beta_{1}=0$ for the sake of identifiability:

(3) Y=Xβ+ϵ,

where **X** is the design matrix in which the k(k>1)-th row $X_{k}=(\ldots ,-1,\ldots ,1,\ldots )$: the *i*(*k*) position is –1 and the *j*(*k*) position is 1. **Y** is the column vector of contig distance observations, and ϵ is the column vector of errors. In this way, thousands of links are compressed into $\sum_{\left(i,j\right)}K_{ij}$ observations, without losing any linking mode.

Any parameter estimate of the above regression model gives a solution of the order as well as positions of the contigs within a scaffold. The least square (LS) estimate minimizes the sum of residual squares, which measures the goodness of a scaffold. Since our compression strategy may retain multiple distances for each contig pair, a small portion of observations would be mistaken and we need to eliminate them from the estimate. This requires a robust regression method with a high breakdown value (Huber, 1996). That is, the estimates would not break down even in the presence of a fair portion of outlier observations. To achieve the goal, we adopt a revised least trimmed squares (LTS) estimate (Li, 2005; Rousseeuw and Leroy, 2003), which tries to minimize the trimmed proportion while minimizing the sum of trimmed squares:

(4) $$\underset{\hat{\beta };I^{*}\subseteq I}{\text{min}}\left[\sum_{k\in I^{*}}w_{i,j}^{k}{\left({\hat{y}}_{i,j}-y_{i,j}^{k}\right)}^{2}+|I\;\backslash \;I^{*}|\right],$$

where ${\hat{y}}_{i,j}={\hat{\beta }}_{j}-{\hat{\beta }}_{i},\;I=\{k:i(k),j(k)\in V_{G}\}$ denotes the sample space containing all compressed observations on $V_{G}$, and |·| denotes the size of the set ·. Algorithm 1 gives the pseudo-code of the Weighted Least trimmed Squares (WLTS) algorithm. The algorithm iteratively selects a size-decreasing subset containing the smallest residuals under current estimate till all residuals of selected samples are below the given threshold MaxError, which is also an adjustable parameter in RegScaf. At the end, the positions estimated based on the final selected subset will be output. Moreover, the regression procedure gives the confidence intervals of all gaps (Supplementary Note S6) in the output of RegScaf.

The parameter value of MaxError is selected by the following consideration. In an ideal case, all outliers have been filtered out from the final selected subset so that all residuals should satisfy the normal distribution assumption. Remember that a compressed observation is defined by the median of a clustered linking distances. According to the central-limit theorem of median (Shao, 1999), the standard deviation of a compressed observation should be about $\sqrt{\frac{\pi }{2n_{ij}^{k}}}\sigma_{b}$. We consider a typical scenario, where the standard deviation *σ_b_* of the insert-size takes 500 and the cluster size $n_{ij}^{k}$ takes 150, then about 95% residuals would be within two standard deviations, namely, $2\sqrt{\frac{\pi }{2n_{ij}^{k}}}\sigma_{b}\approx 2\times \sqrt{\frac{3.14}{2\times 150}}\times 500\approx 102.33$. Thus, we set the default value to be 100. Since the cluster size decreases as the number of iterations increases, we increase MaxError by 50 in each iteration.

**Algorithm 1: The WLTS algorithm in RegScaf.**

**Input**: The whole sample space **I** on subgraph **G**; MaxError.

**Output**: The final estimate $\hat{\beta }$.

Extract **Y**, **X**, **W** over **I** ;

set $I^{\left(0\right)}=I$; n=|I|; ${\hat{e}}^{\left(0\right)}=(0,\ldots ,0,10000)$; t = 0;

**while** $\max({\hat{e}}^{\left(t\right)}){|}_{I^{\left(t\right)}}>\mathrm{MaxError}$  **do**

Compute the WLS estimate over the current sample space $I^{\left(t\right)}$:

${\hat{\beta }}^{\left(t\right)}=[{\left(X^{T}WX\right)}^{-1}X^{T}WY]{|}_{I^{\left(t\right)}}$ ; and the errors:

$${\hat{e}}^{\left(t\right)}=X{\hat{\beta }}^{\left(t\right)}-Y;$$

extract $I^{\left(t+1\right)}\subseteq I$, where $I^{\left(t+1\right)}$ contains only the (n−t)

samples with the minimum errors in **I**;

update the edge set of **G**: $E_{G}^{\left(t\right)}=\{\left(i(k),j(k)\right):k\in I^{\left(t+1\right)}\}$;

**if *G*** *is not connected on* $E_{G}^{\left(t\right)}$  **then**

break **G** into connected components: G=∪subG

**for** *each subG in* ***G* do**

go to the orientating procedure to scaffold contigs in subG

return;

t+=1

$$\hat{\beta }={\hat{\beta }}^{\left(t\right)};$$

### 2.6 Extending scaffolds iteratively and polishing final scaffolds

Since the linear equations do not require contigs to be non-overlapping, RegScaf further detects and splits tangled scaffolds in the regression result into *super-contigs* by selecting the most linked neighbor at each branch (Supplemental Note S7). In each super-contig, contigs are linearly positioned with adjacent overlaps no more than a given length. To obtain longer scaffolds, RegScaf repeats the above scaffolding pipeline by replacing initial contigs with current super-contigs. Super-contigs get longer as the number of iterations increases; meanwhile the number of reliable links decreases and we should stop when links are not sufficient for constructing longer scaffolds. We suggest that users set the iteration parameter *k* = 3 in most situations. If time permits, users can attempt more iterations till the improvement of the new iteration is negligible.

In the end, we polish the final scaffolds by merging overlapping contigs. If the estimated positions of two adjacent contigs overlap within the tolerable range, we perform a local alignment between their overlapping sequences and merge the matched ones.

### 2.7 Data accessibility

The simulation data used for *Escherichia coli* and *Caenorhabditis elegans* are available in our github site at https://github.com/lemontealala/RegScaf.git. GAGE benchmarking data are available at http://gage.cbcb.umd.edu/data. The raw sequencing data for pika genome are available in Genome Sequence Archive (GSA) under the accession numbers ranging from CRX003869 to CRX003876, or at https://ngdc.cncb.ac.cn/search/?dbId=&q=pika. The PacBio data for *E. coli* can be downloaded from https://github.com/PacificBiosciences/DevNet/wiki/E.-coli-Bacterial-Assembly.

## 3 Results

### 3.1 Accurate gap estimates on both synthetic and real data

We first assessed RegScaf on two synthetic datasets and compared it with five off-the-shelf scaffolders: SSPACE, BESST (Sahlin *et al.*, 2012, 2014), SOPRA, Opera-LG and SOAP2. Supplementary Table S7 shows several key parameters used in the reported experiments of RegScaf. Scaffolding results were evaluated using QUAST (Mikheenko, 2018). Besides, we extracted the gap error, which is equal to the gap distance on reference genome minus the gap distance on scaffolds, from QUAST results and computed RMSEs (Root Mean Square Error) to evaluate the accuracy of gap estimates. We also compared the number of extremely abnormal errors in gap estimates, which are defined as estimates with residuals larger than 1000 bp. This threshold is used in the metric ‘#scaffold gap ext. mis.’ (scaffold gap extensive misassemblies) in QUAST Manual at http://quast.sourceforge.net/docs/manual.html. We took two genomes of model organisms: *Escherichia* *coli* (strain K-12 MG1655) and *Caenorhabditis* *elegans* (WBcel235, Chr I-VI) as references and simulated several paired-end Illumina libraries using ART (Huang *et al.*, 2012) (Version 2.5.8). As performances of different scaffolders depend on preliminary contigs, we conducted our tests using two different versions of contigs, referred to as A and B. Version A of preliminary contigs was generated by SOAP2 and its extended version B was obtained by running GapCloser on SOAP2 results.

**Fig. 3.**
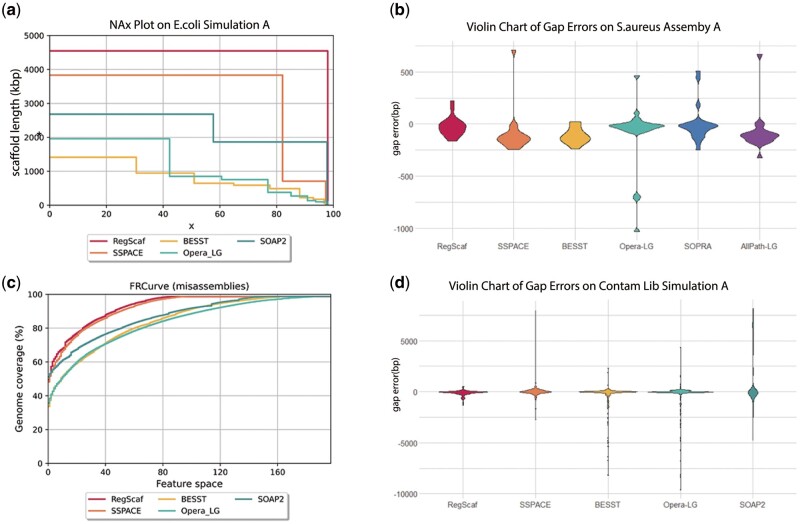
RegScaf outperforms current scaffolders in accuracy. (**a**) NAx plot from QUAST output for five scaffolders on the *E. coli* simulation A. Nx is the length for which the collection of all scaffolds of that length or longer covers at least *x*% of the assembly, while NAx is a corrected version of Nx. QUAST breaks the scaffolds into aligned blocks and calculates the Nx of the blocks, denoted as NAx. The line chart shows NAx values as *x* varies from 0 to 100%. At x=0, from top to bottom, the curves correspond to RegScaf, SSPACE, SOAP2, Opera_LG, and BESST. In this simulation, only RegScaf completed the assembly in one scaffold with no misassembly. (**b**) The violin plot of gap error distributions for results on the *S. aureus* assembly A. The gap_error=EstimateGapSize−RealGapSize, where gap sizes are extracted from QUAST results. Results from both RegScaf and BESST have no outlier while RegScaf shows a smaller bias from 0. (**c**) The FRCurve plot from QUAST output for the PE-contaminated simulation B on *C. elegans*. The *x* value (Feature space) is the total maximum number of features (misassemblies) in the scaffolds. The *y* value (Genome coverage %) is the total number of aligned bases in the scaffolds, divided by the reference length. At x=80, from top to bottom, the curves correspond to RegScaf, SSPACE, SOAP2, BESST, and Opera_LG. RegScaf gets the least misassemblies while SSPACE follows closely. (**d**) The violin plot of gap error distributions for results on PE-contaminated simulation B on *C. elegans*. RegScaf evidently outperforms other scaffolders by substantially reducing extremely abnormal gap errors

Supplementary Table S1 shows the scaffolding statistics of the simulation results. In the case of *E. coli*, based on contig version A, only RegScaf completed the single chromosome with no misassembly (Fig. 3a). Based on the version B in which contigs are extended by GapCloser, three scaffolders reached one complete chromosome: SSPACE, Opera-LG and RegScaf, while RegScaf achieved the least misassembly and the longest NA50. NA50 is a measure on corrected N50 in QUAST: if breakpoints occur when aligning assembled scaffolds to a reference genome, QUAST breaks the scaffolds into aligned blocks and calculates the N50 of these blocks, which is the so-called NA50. In the case of *C. elegans*, RegScaf yielded the least misassembly and the largest NA50 using contig version A, the second least misassembly and the second largest NA50 using version B. In terms of scaffold gap extensive misassemblies and RMSEs, RegScaf reached the least on both versions.

We also assessed RegScaf on three GAGE benchmark datasets: *Staphylococcus* *aureus*, human chr14 and *Bombus* *impatiens*. We selected some relatively high quality contig versions as follows: contigs from Allpaths-LG (Butler *et al.*, 2008) and SOAP2 for *S. aureus*, contigs from Allpaths-LG and CABOG for human chr14, but only one version from SOAPdenovo for *B. impatiens*. Supplementary Table S2 displays the QUAST results for the GAGE datasets. Overall, RegScaf obtained more competitive scaffold length with fewer scaffold misassemblies in most cases. Moreover, RegScaf yielded the least scaffold gap extensive misassemblies and the least RMSEs on most datasets. The violin plot in Figure 3b also shows RegScaf’s accuracy of gap estimates: only RegScaf and BESST have no extreme errors while the former is overall less biased.

### 3.2 Precise reconstruction of tandem repeat regions

One of the greatest challenges in genome assembling is the widespread repeats in genome, among which is a common type tandem repeat. The main difficulty brought by tandem repeats is that the read alignments against homologous regions will induce multiple modes in the position differences between contigs, making it challenging to detect correct ones for existing scaffolders. RegScaf first identifies those multiple modes by grouping links and then selects the more global-supported mode by the robust regression, thus constructing more accurate scaffolds.

From the assessment of the scaffolding results in the *S. aureus* assembly using the contig version A, we found a tandem repeat region and then examined the scaffold resulted from different scaffolders corresponding to this region. SSPACE, Allpaths-LG and SOPRA all reported an abnormal gap estimate, while BESST failed in reconstructing this region and Opera-LG reconstructed this region but dropped out a crucial interior contig. Notably, only RegScaf reconstructed the correct scaffold with an accurate gap estimate. We explain how RegScaf resolves tandem repeats in this instance.

**Fig. 4.**
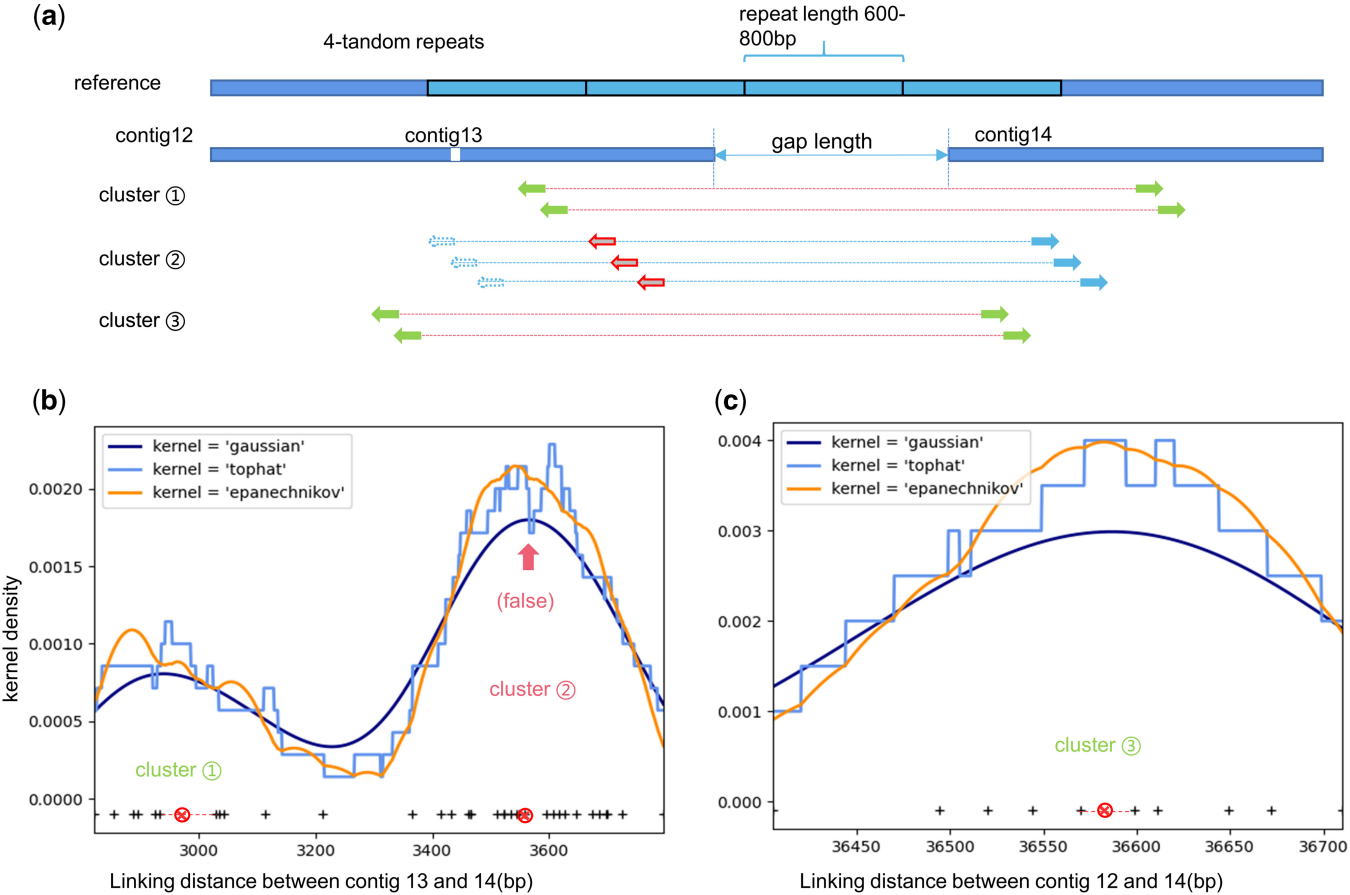
A reference sequence contains a 4-tandem repeat regions (sky-blue consecutive boxes), and three contigs are assembled for this sequence. Mapping reads between contigs are grouped into three clusters, in which links from cluster 2 are false because reads from the edge of contigs (dotted sky-blue arrows) are mis-aligned to its homologous region (red unfilled arrows). Mis-alignments lead to larger observations on linking distances in cluster 2. (**b**) The kernel density of the distance observations on contigs 12 and 13 shows two peaks. The density curve with small steps corresponds to the kernel “tophat”, the smoothest curve corresponds to the kernel “gaussian”, and the slightly wiggled curve corresponds to the kernel “epanechnikov”. The linking distance data are presented in points at the bottom, where the medians are marked by two circles. The lower peak corresponds to the true cluster ①; the higher peak corresponds to the false cluster ②, which current scaffolds are more likely to use to estimate the gap between contig 13 and 14. (**c**) The kernel density of the distance observations from contigs 12 and 13 shows only one peak, which corresponds to the only cluster between contigs 12 and 14. This observation helps RegScaf identify and remove the false observation ② and obtain more accurate scaffold. The three curves correspond to the kernel “tophat”, “gaussian”, and “epanechnikov” as in (b).

As shown in Figure 4, the reference sequence contains a 4-tandem repeat region. Three contigs are assembled for this region and their mapped paired reads are grouped into three clusters.

- Cluster ①: Two reads of the pair are both correctly mapped to contig 13 and contig 14. Linking distances calculated from this cluster are substantially correct so the median is close to the true value;
- Cluster ②: The left read (the dotted sky-blue arrow) which should map to the junction of contig 12 and 13 is mis-mapped to a homologous region (the red arrow) on contig 13. Linking distances calculated from this cluster are larger than the true value and the difference is about a repeat length (600 bp);
- Cluster ③: Two reads of the pair are both correctly mapped to contig 12 and contig 14. Linking distances calculated from this cluster are also substantially correct so the median is close to the true value.

The advantage of RegScaf is highlighted in this example. Since the misleading links between contigs 12 and 13 overwhelm correct ones, scaffolders using greedy or local strategies, like SSPACE and SOAP2, are bound to fail in detecting them. Scaffolders which only retain one observation for one contig pair, like Opera-LG and SOPRA, will also ignore the weaker signal of linking mode ① and position contigs according to the stronger linking mode ②. Nevertheless, considering the three linking modes globally, RegScaf is able to identify and remove the false mode ②, thus positioning contigs accurately.

We also assessed the performance of RegScaf by the Repeat-aware Evaluation framework (Mandric *et al.*, 2018), using the *S. aureus* dataset from GAGE. The procedure and code of evaluation follows the guideline at https://github.com/mandricigor/repeat-aware. Detail can be found in Supplementary Note S9 and Supplementary Table S5. Compared with SSPACE, BESST, SOPRA and Opera-LG (with or without repeat contigs), RegScaf performed better in the number of correct links, sensitivity, PPV and F-scores. These results demonstrate the advantage of RegScaf in reconstructing repeat regions.

### 3.3 Robustness to PE-contaminated libraries

Although the base quality of SGS reads is quite high (over 99.9%), insert-size errors occasionally occur in library preparation. In this case, a mate-pair library whose insert-size is designed larger than 2kbp is contaminated by a proportion of short-insert paired-end reads, resulting in a PE-contaminated library. The insert-size distribution of a PE-contaminated library is usually in a bimodal form: one peak stands around the designed size and another stands around a few hundred bps.

As we have mentioned in Section 2, our method adopts the robust estimate based on clustered linking modes, which is able to handle a fair proportion of contaminated observations. To verify this, we simulated two contaminated libraries on *C. elegans* genome, each of which composes 40% wrong insert-size reads, and conducted genome assembly. The results in Supplementary Table S3 show RegScaf obtains a comparable scaffold length with high accuracy: it yields the second largest and the largest NA50 using the two contig versions, respectively. As shown in Figure 3c and d, RegScaf also yielded the least misassemblies and the most accurate gap estimates. Notably, the gap error distributions of other methods have long tails, which means many gaps estimates are severely biased. In contrast, such extremely biased gap estimates have been substantially reduced in RegScaf.

### 3.4 Outperformance on a large genome

As the sequencing costs drops, the sequencing of many non-model organisms becomes realistic. Consequently, more insights can be gained from the comparative genomic studies. For example, to understand how pika or *O. curzoniae*, adapts to the environment of high altitude and low oxygen, our collaborators from Kunming Institute of Zoology, CAS, initiated a sequencing project of pika, the complete genome size of which is about 2.5 Gb. The sequencing data contain 13 Illumina libraries including 7 PE and 6 MP libraries. All 13 libraries were used in *de novo* assembly while the scaffolding step only used 85× sequencing reads including the 6 MP libraries and the longest PE library.

We generated preliminary contigs by cutting a *de novo* assembly from SOAP2. Next, we conducted scaffolding by SSPACE and RegScaf, respectively, and their results were compared in Table 1. Both methods reduce the scaffold number to less than one third of the number of initial contigs. The scaffold N50 value is 0.53 Mb in SSPACE compared to 2.4 Mb in RegScaf, an increase of more than 4-folds. The BUSCO value (Simão *et al.*, 2015), an index indicating the biological completeness of an assembly, is increased from 93.5% to 95.3% by RegScaf. Notably, due to the more accurate gap estimates, Regscaf is able to recognize more overlapping contigs, and the contig merging doubles the contig N50 from 27.3 to 57.5 kb.

**Table 1.** Scaffolding statistics on the *O. curzoniae* genome

	Contig N50 (kb)	No. of contigs	scaffold N50 (kb)	No. of scaffolds	BUSCO (%)
Original	27.3	162408			
RegScaf	**57.5**	100758	**2403.4**	41388	95.3
SSPACE	30.8	150970	531.4	50629	93.5

Note: The best N50 indices are marked in bold.

### 3.5 Adaptability to TGS platforms

We show the adaptability of the regression model to sequencing data from other platforms by an example of PacBio long reads (Roberts *et al.*, 2013). *Escherichia* *coli* PacBio data and reference genome were downloaded from https://github.com/PacificBiosciences/DevNet/wiki/E.-coli-Bacterial-Assembly. We also obtained a corrected version of the PacBio dataset using LoRDEC (Salmela and Rivals, 2014). Three simulated Illumina libraries are used to generate contigs by SOAP2 and reads mapping were carried out by BLASR (Chaisson and Tesler, 2012). RegScaf was compared with two hybrid scaffolders: SSPACE-LongRead (Boetzer and Pirovano, 2014) and LRScaf (Qin *et al.*, 2019) (see Supplementary Table S4). Both RegScaf and LRScaf completed the genome assembly in one scaffold using either uncorrected long reads or corrected long reads, while SSPACE-LongRead ended up with dozens of scaffolds. In addition, both RegScaf and LRScaf introduce no misassembly in the final scaffold, except the trivial difference at the breakpoint of the circular genome.

## 4 Discussion

In shotgun sequencing, the scaffolding step plays a significant role in the accuracy and the continuity of final assemblies. Nevertheless, scaffolding is still a difficult problem regarding the widespread repeats in genomes. In the presence of incomplete preliminary contigs, reads from unassembled regions may be aligned to homologous sequences and be mistakenly used to position contigs. Only if we are able to identify such misalignments leading to incorrect inter-contig distances, can we resolve such repeat regions accurately in scaffolding. To our best knowledge, RegScaf is the first scaffolder that takes into account multiple modes of linking distances between contigs, for the purpose of including the correct ones with a better chance, and selects the optimal solution according to a global measure of consistency.

In measurement problems, robust regression methods are generally accredited for their high breakdown value as well as accuracy, which are exactly what we need in genome assembly. Our method takes full advantage of the statistical method by formalizing scaffolding as a genomic position estimate problem in a regression model. In principle, this regression model can apply to any sequencing platforms, such as TGS, 10× sequencing and even optical mapping (Schwartz *et al.*, 1993) data as long as the linking distance can well be defined. In this article, we applied the regression method to SGS and TGS data, and the preliminary efforts showed its feasibility and reliability in contig ordering and gap estimation. We will expand RegScaf’s adaptability by adding data-processing scripts for other sequencing platforms in the future.

Although the cost of sequencing decreases over time, the entire workload of a genome project is still quite intensive. It is of great value to improve the accuracy and continuity of assemblies using existing the huge amount of SGS data. As shown by the pika genome, RegScaf improved the N50 values reliably and substantially.

Moreover, the regression model proposed in this article can be applied to evaluating existing scaffold assemblies. For any given scaffold, we can plug its contig position estimates into the regression model and compute residuals for all linking distance samples. Samples with abnormally large residuals are then marked as outliers. If the connectivity of a contig subgraph is broken after excluding those outliers, it indicates that the broken junction is suspicious under the current estimate. Indeed, an assessing module based on the regression model, can be found in the program at https://github.com/lemontealala/RegScaf.git/pipeline_assess.sh.

Sequencing data from one generation to the next have brought opportunities as well as challenges to assemblers. In assembling, an accurate scaffold can be treated as a ‘super-contig’ if all gaps are positioned with accuracy of high reliability. It is our hope that the proposed robust regression approach can make assembly, a cornerstone for downstream genomic analysis, more reliable.

## Supplementary Material

btac174_Supplementary_Data
