# Supplementary material for: RegScaf: a regression approach to scaffolding
Source: Bioinformatics. 2022 Mar 25;38(10):2675–82. doi: 10.1093/bioinformatics/btac174 (PMC9326850; doi:10.1093/bioinformatics/btac174)
Supplement: btac174_Supplementary_Data [file btac174_supplementary_data.zip › SuppleMentary_Materials.pdf]

Supplementary Note S1: Filtering high-coverage areas from mapping results High-coverage areas are filtered out for SGS data and TGS data separately. For SGS data, high-coverage areas are marked using scripts from BAUM. We first compute the mapped coverage of the whole preliminary contigs by reads; areas on contigs with coverage $> 1.5\times$ average will be marked as high-coverage and be filtered out when processing mapping results into links. For TGS data, average coverage is computed for each contig separately. Contigs with coverage higher than 80% quantile of all will be excluded when processing links.

Supplementary Note S2: Proof of maximum likelihood solution in the orientating model Assume that each link is misdirected with an equal probability p independently, with $p \ll 1$. The likelihood of a given orientation $\mathbf{D}$ is

$$\begin{aligned} Pr(\mathbf{D} = (D_1, D_2, \ldots, D_m)) &= (1-p)^{\sum_{r:d_{ij}^r = D_i D_j}} p^{\sum_{r:d_{ij}^r \neq D_i D_j}} \\ &= (1-p)^R \left(\frac{p}{1-p}\right)^{\sum_{r:d_{ij}^r \neq D_i D_j}}, \end{aligned} \tag{1}$$

where $R = \sum_{r:d_{ij}^r = D_i D_j} + \sum_{r:d_{ij}^r \neq D_i D_j}$ denotes the sum of all links in $\mathbf{G}$, which is a constant. So maximizing the likelihood $Pr(\mathbf{D})$ is equivalent to minimizing the total discordant link counts: $\mathbf{T}(\mathbf{D}) \triangleq \sum_{r:d_{ij}^r \neq D_i D_j}$. For a given contig pair (i, j), considering that D_i, D_j both take value from $\{1, -1\}$, we have

$$\#\{r : d_{ij}^r \neq D_i D_j\} = \begin{cases} b_{ij} \triangleq \#\{r : d_{ij}^r = -1\} & \text{if } D_i = D_j, \\ a_{ij} \triangleq \#\{r : d_{ij}^r = 1\} & \text{if } D_i \neq D_j. \end{cases} \tag{2}$$

The total discordance can be rewritten as:

$$\begin{aligned} \mathbf{T}(\mathbf{D}) &= \sum_{i \neq j} \frac{1}{2}[(1 - D_i D_j) a_{ij} + (1 + D_i D_j) b_{ij}] \\ &= -\sum_{i \neq j} \frac{1}{2}(a_{ij} - b_{ij}) D_i D_j + \frac{1}{2} \sum_{i \neq j} (a_{ij} + b_{ij}) \\ &= \mathbf{H}(\mathbf{D}) + \frac{1}{2} \sum_{i \neq j} (a_{ij} + b_{ij}) \end{aligned} \tag{3}$$

Since the second term in (3) is irrelevant of $\mathbf{D}$, we have proved that minimizing $\mathbf{H}$ is equivalent to minimizing $\mathbf{T}(\mathbf{D})$ over all possible orientation assignments, which is also equivalent to maximizing the likelihood (1).

Supplementary Note S3: Orientating algorithm RegScaf adopts a heuristic algorithm to orientate contigs in the subgraph $\mathbf{G}$. The algorithm first initializes the orientation assignment $\mathbf{D}$ by a weight-decreasing depth-first search; then it iteratively optimizes $\mathbf{H}$ node by node.

Before the initialization, we first assign each edge (i, j) a weight $p_{ij} = max(a_{ij}, b_{ij})$ along with a flag D_{ij}, where $D_{ij} = 1$ if $a_{ij} \geq b_{ij}$; $D_{ij} = -1$ if $a_{ij} < b_{ij}$. Then we start from the first vertex whose orientation has been set to be positive, and visit all other vertices in a depth-first order. When visiting a new vertex, its orientation D_{new} is determined by the orientation of its precursor D_{pre} and their edge flag $D_{pre,new}$: $D_{new} = D_{pre}D_{pre,new}$. Once the vertex has been visited, its heaviest unvisited neighbor is the next to be visited. The initialization is completed when all vertices have been visited, that is, have been assigned with an initial orientation. In fact, many traversing algorithms, such as a simple depth-first traversing algorithm, a broad-first traversing algorithm, or the minimum generating tree algorithm can be applied to initialization.

Then we optimize the initial orientation $\mathbf{D}^{(0)}$ node by node. In each iteration, we first compute the change of $\mathbf{H}$ by reversing each vertex separately: $\Delta\mathbf{H}_i = \mathbf{H}(\mathbf{D}_{-i}) - \mathbf{H}(\mathbf{D})$, where $\mathbf{D}_{-i} = (D_1, \ldots, -D_i, \ldots, D_m)$. Second, we select the vertex which will bring the largest reduce of $\mathbf{H}$ and reverse its orientation. If reversing any single vertex will increase $\mathbf{H}$, the optimization step is terminated. The optimizing algorithm is as follows:

- Step 0: k=0, initialize $\mathbf{D}^{(0)}$;
- Step 1: Compute $\Delta\mathbf{H}_i(\mathbf{D}^{(k)}) = \mathbf{H}(\mathbf{D}_{-i}) - \mathbf{H}(\mathbf{D}), i = 1, 2, \ldots, n$;
- Step 2: If $\min_{1\leq i\leq m} \Delta\mathbf{H}_i(\mathbf{D}^{(k)}) \leq 0$, let $\mathtt{min_i} = arg\min_i \Delta\mathbf{H}_i$, $\mathbf{D}^{(k+1)} = \mathbf{D}^{(k)}_{-\mathtt{min_i}}$, and $k = k + 1$, go to Step 1. Otherwise, let $\hat{\mathbf{D}} = \mathbf{D}^{(k)}$, output $\hat{\mathbf{D}}$ and stop.

Supplementary Note S4: Computing linking distance from alignment of SGS paired read Since we denote s_i^r as the mapping coordinate of the outer end of the read r on contig i, regardless of the

read type, the computing of linking distance is the same for the pair-end reads with it for the mate-pair reads. As shown in Fig S1, since all contigs have been adjusted into the consistent orientation, only two cases may occur in the read mapping results. Case 1: contig i is upstream of contig j; case 2: contig j is upstream of contig i.

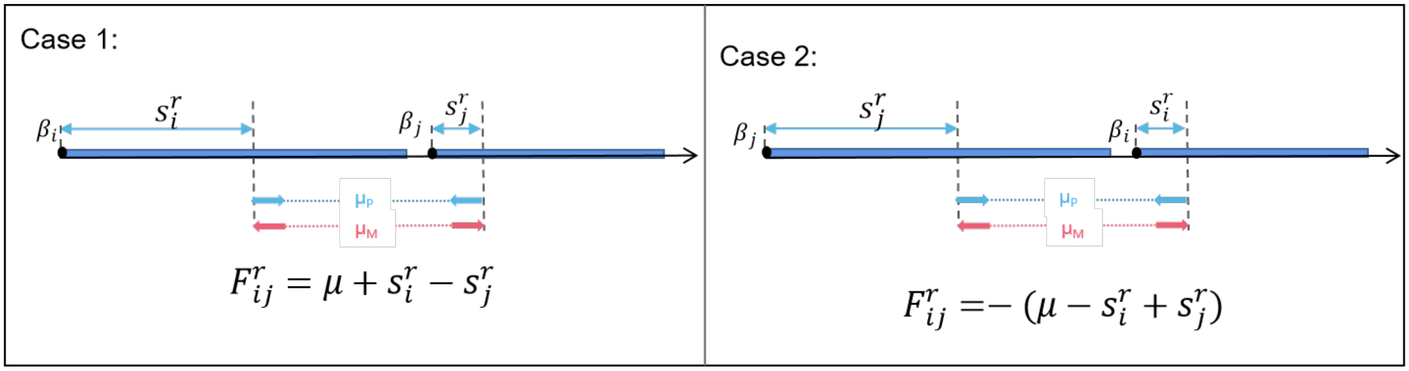

Figure S1. An illustration of linking distance calculation from alignment of SGS paired reads

Supplementary Note S5: Computing linking distance from alignment of TGS long read

Assume two segments of the long read r are mapped to two contigs i, j separately. Let qs_i^r denote the alignment start coordinate on the long read and cs_i^r denote the alignment start on contig i. The linking distance can be calculated by:

$$F_{ij}^r = qs_j^r - qs_i^r + cs_i^r - cs_j^r.$$

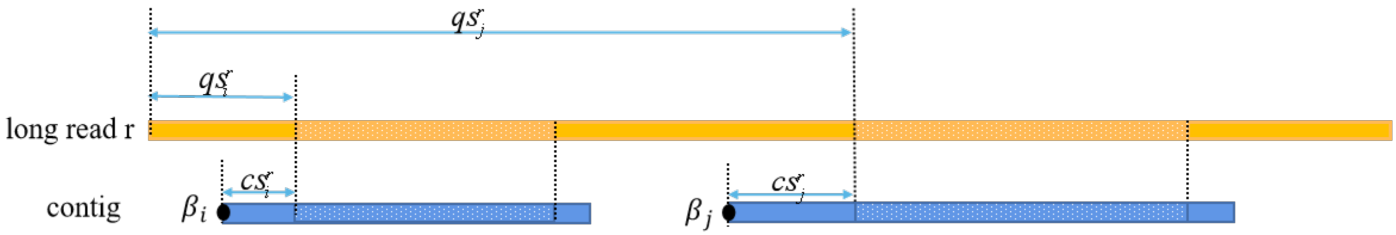

Figure S2. An illustration of linking distance calculation from alignment of TGS long read

Supplementary Note S6: Empirical confidence intervals of gap estimates Once the regression procedure has given the estimates of contig positions, RegScaf obtains the estimates of all gap distances immediately. Denote **R** be the rearrangement matrix which transforms the unit vector into the contig order in final scaffold and extracts the gap length between adjacent contigs. For example, assume the

contig order in final scaffold is $\{3, 1, 5, 2, 4\}$, then

$$\mathbf{R} = \begin{pmatrix} 1 & 0 & -1 & 0 & 0 \\ -1 & 0 & 0 & 0 & 1 \\ 0 & 1 & 0 & 0 & -1 \\ 0 & -1 & 0 & 1 & 0 \end{pmatrix}.$$

Then the gap estimate can be computed in a matrix form:

$$\hat{\mathbf{G}} = \mathbf{R}\hat{\boldsymbol{\beta}} - \mathbf{L}, \tag{4}$$

where $\mathbf{L} = (len_1, len_2, \ldots, len_{m-1})^T$ denotes the vector of the length of the first $m-1$ ordered contigs.

Moreover, we can give an empirical confidence interval for each gap estimate. We first obtain the covariance matrix of the final $\hat{\boldsymbol{\beta}}$ and then multiply it by the rearrangement matrix $\mathbf{R}$:

$$Cov(\hat{\mathbf{G}}) = \mathbf{R}Cov(\hat{\boldsymbol{\beta}})\mathbf{R}^T. \tag{5}$$

Furthermore, denote $\boldsymbol{\Lambda} = (X^T\mathbf{W}X)^{-1}X^T\mathbf{W}$ be the coefficient matrix of the final $\hat{\boldsymbol{\beta}}$ in the WLTS procedure, that is, $\hat{\boldsymbol{\beta}} = \boldsymbol{\Lambda}\mathbf{Y}$, hence

$$Cov(\hat{\boldsymbol{\beta}}) = \boldsymbol{\Lambda}Cov(\mathbf{Y})\boldsymbol{\Lambda}^T. \tag{6}$$

We plug (6) into (5) and substitute the $Cov(\mathbf{Y})$ with the sample variance $\mathbf{S}(\mathbf{Y})$, which is a diagonal matrix formed by the sample variances of linking distances in each cluster:

$$Cov(\hat{\mathbf{G}}) = \mathbf{R}\boldsymbol{\Lambda}\mathbf{S}(Y)\boldsymbol{\Lambda}^T\mathbf{R}^T. \tag{7}$$

Afterwards the diagonal elements of $Cov(\hat{\mathbf{G}})$ are extracted as the variances of gap estimates for the final scaffold. Therefore, an empirical confidence interval of the i-th gap is:

$$\left[\hat{G}_i - 2\sqrt{(Cov(\hat{\mathbf{G}}))_{ii}},\ \hat{G}_i + 2\sqrt{(Cov(\hat{\mathbf{G}}))_{ii}}\right],$$

where $(\cdot)_{ii}$ denotes the i-th diagnal element of the matrix $\cdot$. Variances are also output in 'Final_n.txt' in RegScaf results.

Supplementary Note S7: Untangling the regression result into super-contigs Due to the widespread repeats in genome, contigs from remote regions, sometimes even from different chromosomes, could be linked by mis-aligned reads. Errors in libraries preparation and contig generation will also lead to such tangles. Although the WLTS procedure can detect and remove misleading linking modes which contradict with most relative observations, it cannot identify those conflict-free ones. For example, if only one linking mode presents between two contig subsets, the WLTS procedure will reserve them in one connected subset, resulting the so-called tangled scaffold. SOPRA detects and splits the tangled scaffold by the contig density profile, while RegScaf adopts a more directive approach by selecting the most linked neighbor at each branch.

Provided that the sequencing coverage is approximately uniform, the number of links between adjacent regions should be significantly larger than that between remote regions. Therefore, we can utilize adjacent link information to split the tangled regression result into separate subsets, each corresponding to a super-contig. In each regression result, RegScaf first sorts contigs by their estimated start coordinates. Then starting from the leftmost contig, it moves a length-fixed window over to pick the most possible successor one by one. The window starts from $\texttt{3MAXSD}$ bp left of the current contig end and extends rightward for $\texttt{MAXIN} + \texttt{6MAXSD}$ bp, where $\texttt{MAXSD}$ and $\texttt{MAXIN}$ are set to the largest variance and the largest insert-size, respectively. In this window, RegScaf will pick the contig which has the most links with the current contig to join the current subset; then it moves the window to the end of the selected contig and continue finding the next most linked neighbor. If no linked contig is found in the window, selection for the current subset is terminated; those unselected contigs are left for another super-contig. This procedure is repeated until every contig has been placed in a subset. Ultimately contigs in each subset are in a linear arrangement with overlaps no more than $\texttt{3MAXSD}$ bp, forming an untangled super-contig.

Supplementary Note S8: Parameters used in experiments The software includes several parameters. k is the number of iterations, m is the threshold which controls the minimum link count for

Table S1_Simulations:
E.coli A: -k 3, -m 20, -M 100 (iter2/3: 150)
E.coli B: -k 3, -m 20, -M 100 (iter2/3: 150)
C.elegans A: -k 4, -m 22, -M 100 (iter2/3/4: 200)
C.elegans B: -k 3, -m 22, -M 100 (iter2/3: 200)
Table S2_GAGE:
S.aureus A: -k 3, -m 12, -M 100 (iter2/3: 200)
S.aureus B: -k 3, -m 12, -M 100 (iter2/3: 200)
Human_chr14 A: -k 3, -m 6, -M 100 (iter2/3: 200)
Human_chr14 B: -k 3, -m 12, -M 100 (iter2/3: 200)
B.impatiens: -k 3, -m 12, -M 100 (iter2/3: 150)
Table S3_C.elegans_ContamLib
A: -k 3, -m 22, -M 100 (iter2/3: 150)
B: -k 3, -m 12, -M 100 (iter2/3: 150)
Table S4_Ecoli PacBio3GS
A(RawdataScaf): -k 3, -m 20 (iter2/3: 30) -M 100 (iter2/3: 150)
B(EcoliPolishRef): -k 3 , -m 20 (iter2/3: 30) -M 100 (iter2/3: 150)
Table S5_Saureus Repeat-Aware
-k 3, -m 6 -M 100 (iter2: 150; iter3: 200) -s 100
Table 1_Ochotona.curzoniae genome
-k 2 -m 12 -M 100 (iter2: 150)

Table S7. Parameters used in experiments

defining an edge in the scaffolding graph, and M is the threshold `MaxError` which controls the maximal absolute value of residuals allowed in the final regression. According to the design of the pipeline and our experiences, it will be more efficient to set the value of `MaxError` more stringent in the first iteration than those in the subsequent ones as shown in the parentheses. Table S7 shows the parameter values used in the reported experiments. They can serve as references for other scaffolding situations.

Supplementary Note S9: Assessing scaffolding results using Repeat-aware Evaluation
Repeat-aware Evaluation framework (Mandric *et al.* 2018) assesses scaffolding results by taking into account multiple locations of repeat sequences on the reference scaffolding. It first modifies original contigs by splitting out partial repeats, and then finds a mapping of the inferred scaffolding onto the reference maximizing the number of correct links. To evaluate the performance of RegScaf in the repeat-

aware framework, we carried out an experiment on the S.aureus dataset from GAGE (Salzberg *et al.* 2012). The evaluation was based on the Illumina short-jump library corrected by AllPaths-LG and the preliminary contigs obtained by Velvet.

Following the guideline at `https://github.com/mandricigor/repeat-aware`, we first produced repeat-split contigs and generated the reference scaffolding using build_ref_scaf.py, then we ran five scaffolding tools including RegScaf, SSPACE, BESST, SOPRA, and OPERA-LG on the split contigs. The mapping tool varies from one scaffolder to another depending on their preprocessing procedures: RegScaf and SSPACE used SEME (since we have ready-made scripts preprocessing SEME output into TAB files), BESST and OPERA-LG used BWA (Li and Durbin 2010), and SOPRA used Bowtie2 (Langmead and Salzberg 2012) (since Bowtie2 allows fasta files as input). As last, we implemented the validation.py on the scaffolding results. As shown in Table S5, RegScaf outperformed in the number of correct links, sensitivity, PPV and F-scores. In addition, the result of Opera-LG using the repeat contigs option is slightly better than that without repeat, as reported in (Mandric *et al.* 2018). Thus, the repeat-aware evaluation supported that RegScaf has certain strength of handling repeats in scaffolding.

Supplementary Note S10: The DFS algorithm applied in RegScaf We apply a depth-first-search (DFS) algorithm to obtain all connected subgraphs of the scaffolding graph. Each subgraph corresponds to a subset of contigs that form a scaffold. We implement the algorithm by a recursion whose pseudo-code is shown in Algorithm S1.

Supplementary Fig S3 Fig S3 describes a typical scenario in which missing duplicate regions on contigs lead to mistaken alignment of paired reads. A and B are two copies of a homologous duplicate. The right end of the paired read maps to a correct position. However, the incompleteness of the real source B on preliminary contigs, leads to a mistaken alignment between the left end and the homologous duplicate A. Hence a misleading link is built.

Algorithm S1 The DFS algorithm.

Require: The scaffolding graph **G**;

Ensure: The vertex sets of maximal connected subgraph `All_Subsets`.

```
function DFS(G, node, visit, in_subG_node)
    if visit[node] == 0 then
        in_subG_node.append(node)
        visit[str(node)] = 1
    end if
    all_neighbors = allNeighbors(G, node)
    for neighbor ∈ all_neighbors do
        if visit[neighbor] == 0 then
            in_subG_node = DFS(G, neighbor, visit, in_subG_node)
        end if
    end for
    return in_subG_node
end function
visit = {}
for node ∈ V(G) do
    visit[node] = 0
end for
remained_node = V(G)
All_Subsets = []
while len(remained_node) > 0 do
    node_0 = remained_node[0]
    subG_nodes = []
    subG_nodes = DFS(G, node_0, visit, subG_nodes)
    for node ∈ subG_nodes do
        remained_node.remove(node)
    end for
    All_Subsets.append(subG_nodes)
end while
```

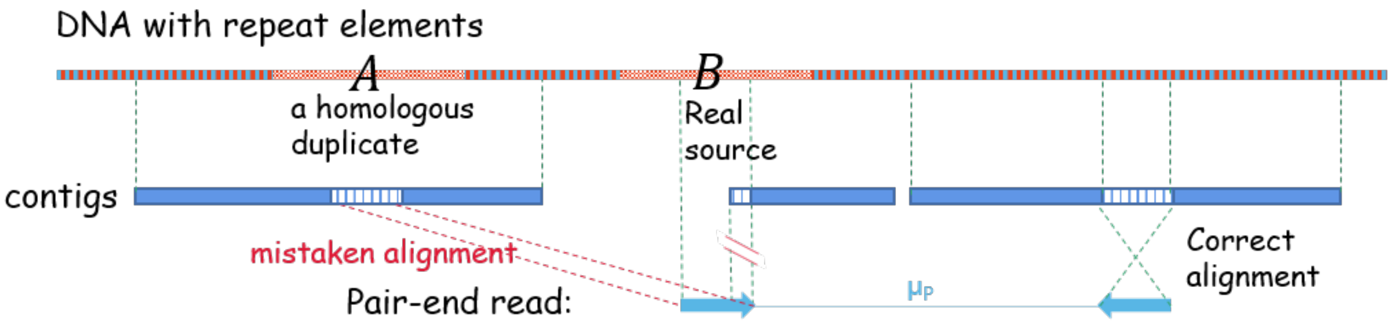

Figure S3. A misleading link caused by missing duplicates in contigs

References

Langmead, B. and Salzberg, S. L. (2012). Fast gapped-read alignment with bowtie 2. **9**, 357–359.

Li, H. and Durbin, R. (2010). Fast and accurate long-read alignment with burrows–wheeler transform. **26**, 589–595.

Mandric, I., Knyazev, S., and Zelikovsky, A. (2018). Repeat-aware evaluation of scaffolding tools. **34**, 2530–2537.

Salzberg, S. L., Phillippy, A. M., Zimin, A., Puiu, D., Magoc, T., Koren, S., Treangen, T. J., Schatz, M. C., Delcher, A. L., Roberts, M., Marcais, G., Pop, M., and Yorke, J. A. (2012). GAGE: A critical evaluation of genome assemblies and assembly algorithms. *Genome Research*, **22**(3), 557–567.
